# Pharmacist-led antibiotic interventions in infectious disease patients: a Pakistani tertiary care antimicrobial stewardship study

**DOI:** 10.1080/20523211.2025.2450017

**Published:** 2025-01-16

**Authors:** Ali Hassan, Naeem Ur Rehman, Sumeira Maqbool, Mehreen Arif

**Affiliations:** a Faculty of Pharmacy, Gomal University, Dera Ismail Khan, Pakistan; b Jinnah Postgraduate Hospital, Lahore, Pakistan; c Shifa College of Pharmaceutical Sciences, Shifa Tameer e Milat University, Pakistan

**Keywords:** Antimicrobial stewardship, antibiotics, clinical pharmacy and clinical pharmacist

## Abstract

**Background:**

Antibiotics are widely used medications among infectious disease patients; therefore, proper monitoring and assessment are critical for ensuring rational use. Antimicrobial stewardship addresses the rational and appropriate use of antibiotics, which reinforces overall health outcomes. Ongoing antimicrobial resistance scenarios are an alarming condition for healthcare, necessitating continued practice of such assessments.

**Objectives:**

To evaluate the use of antibiotics in patients with infectious diseases, implement and evaluate clinical pharmacy interventions that adhere to antimicrobial stewardship protocols.

**Methods:**

A before and after study was designed to evaluate clinical pharmacy and antimicrobial stewardship interventions for infectious disease patients at a tertiary care hospital in Lahore. A Performa was designed for manual data collection. Study first identified the signal of error, implemented intervention and noted post-interventional followups.

**Results:**

102 infectious disease cases were analyzed in total and proposed 136 interventions. Physicians accepted 66% of the interventions (90) and rejected the remaining ones as unjustified. The most accepted intervention was the spectrum-based choice (*n* = 30), followed by de-escalation of dose (*n* = 17). Use of ceftriaxone was very high (54 Pt.), followed by vancomycin (30 Pt.).

**Conclusion:**

Antimicrobial stewardship programmes are critical for any institution's proper health care system. It ensures proper antibiotic outflow to patients, thereby improving their health status. The role of pharmacists in establishing an AMS in a hospital setting is a highly commendable activity that enhances healthcare collaboration and outcomes. Clinical pharmacists should implement such activities to improve patient care.

## Background and introduction

Antimicrobial resistance (AMR) is a health issue that has recently gained attention from different sectors, especially the public health sector due to the rising death rates, which is a worrying sign that calls for action (Naghavi et al., 2024). According to recent data, resistance could lead to up to 10 million yearly deaths by 2050, making it a significant global health hazard. It directly caused 1.27 million deaths in 2019, while it indirectly contributed to 4.95 million deaths, underscoring the urgent need to address this issue (Naghavi et al., 2024; World Health Organization, 2023). To reduce this pattern of resistance, different global actions were taken. The World Health Organization (WHO) has put various regional and global processes in place to reduce drug resistance and minimise its effects, and one of them is the Global Action Plan (World Health Organization, 2016). Despite the implementation of the Global Action Plan and UN sustainability programmes, the fight with AMR has been uncertain, and the main reason for this uncertainty is the uneven implementation and funding of national action plans. However, its implementation has faced challenges in most low- and middle-income countries (LMICs) including Pakistan regarding resources and low human capital (Saleem et al., 2018; Willemsen et al., 2022). Inappropriate antimicrobial prescriptions enhance this situation in many ways, particularly in hospitals where the heavy use of antibiotics on the WHO ‘Watch’ list contributes to rising resistance (Klein et al., 2021; Sulis et al., 2022). Despite several ASPs in LMICs facing challenges such as costs and lack of human resources, new studies argue that new programmes to improve prescription practices could control resistance in Pakistan (Afzal et al., 2024; Haseeb et al., 2023). However, the lack of extensive global data, especially in low-income areas, makes it harder to understand AMR's long-term trends and how well current treatments work. Fixing this problem is necessary to plan future antimicrobial stewardship, infection prevention, and public health initiatives (Cox et al., 2017). The causes of inappropriate antibiotic use are multifaceted and include unnecessary use, inappropriate dosing patterns, choosing an inapt regimen for therapy, improper discontinuation of therapy before optimal treatment, and the use of antibiotics as over-the-counter drugs (Dyar et al., 2014; Ewers et al., 2017). In addition to AMR, inappropriate use of antibacterial agents demonstrates a variety of other health concerns, such as adverse events. A study reported that these events account for 19.3% of all Emergency Department visits for drug-related adverse events, with the highest rates in infants and female patients. The most commonly reported events include allergic reactions (hypersensitivity) and gastrointestinal problems (nausea, vomiting, constipation, and diarrhea) (Shehab et al., 2008). Specific antibiotics such as ampicillin or amoxicillin, clindamycin, third-generation cephalosporins, and fluoroquinolones can cause *Clostridium difficile* infections, which are particularly concerning (Mohsen et al., 2020). Recognising these challenges, the World Health Organization (WHO) has called for responsible antibiotic use through the implementation of antimicrobial stewardship (AMS) programmes.

Antimicrobial stewardship (AMS) refers to the responsible use of antimicrobial agents to combat microbial infections. Responsible use of antibiotics involves selecting the appropriate antibacterial agent to target the specific pathogen causing the infection, administering the antibiotic at the right time, and ensuring the appropriate dosage, route, and method of administration (Dyar et al., 2017). According to Dyar, antimicrobial stewardship is a coherent set of actions designed to use antimicrobials responsibly. Globally, approximately 60% of countries have implemented AMS programmes with varying degrees of effectiveness (Dyar et al., 2017). Multiple studies have documented the effectiveness of AMS programmes in reducing antibiotic use and AMR rates. A 2022 study in the United States found that AMS programmes reduced antibiotic use by 20% and AMR rates by 15% (Yang et al., 2022). European hospitals observed similar positive trends (10% reduction in use, 5% reduction in AMR) and, to a lesser extent, African hospitals (5% reduction in use, 2.5% reduction in AMR) (Ivers & Laxminarayan, 2021). While the specific reduction percentages may vary across these studies, the overall message of effectiveness remains consistent. Despite the success of AMS programmes in reducing antibiotic use and AMR rates worldwide, its implementation in Pakistan remains limited. Pakistani hospitals are now using such programmes to enhance antibiotic utility (Atif et al., 2021; Bin et al., 2015; Khursheed et al., 2023). No doubt there is a need for certain criteria and guidelines, as a paper shows the utter need for AMS Guidelines in Pakistani healthcare to prevent surgical site infections (Khan et al., 2020). A similar Pakistani study argues about the trends of antibiotic prescribing among physicians, which shows a great impact on AMS programmes (Atif et al., 2021). To address the extensive issue of antimicrobial stewardship in Pakistan, this study aimed to evaluate the pharmacist-led investigation of possible intervention in a tertiary care hospital. The study's goal was to assess the use of antibiotics in infectious disease patients, detect a possible signal regarding inappropriate antibiotic use, and then propose relevant interventions to overcome inappropriate use. To address this issue, a before and after study was conducted and achieved effortful success, characterised by improved patient health and a reduction in antibiotic use.

This study's significance highlights the practical approach to antimicrobial stewardship in a hospital setting in Pakistan, where the ratio of such programmes is lower from a global point of view. The study explains the role of a clinical pharmacist in implementing interventions in antibiotic prescribing regimens, as well as the importance of their participation in direct patient care for infectious diseases. Moreover, the study explains the use of before-after studies in implementing stewardship programmes, which is a conventional method of assessing these patterns. Antimicrobial stewardship programmes are essential for combating antimicrobial resistance and improving patient outcomes in hospital settings, and clinical pharmacists play a vital role in implementing and managing these programmes.

## Methodology

### Study setting

This study was carried out at a tertiary care hospital in Lahore, Pakistan from July 2023 to August 2023, considering a two-month analysis.

### Study design

A prospective before and after study design was established for the evaluation and optimisation of antibiotic therapy. Guidelines from the World Health Organization (WHO), Society of Infectious Diseases Pharmacists (SIDP), and ASHP’s book *The Pharmacist’s Guide to Antimicrobial Therapy and Stewardship* were adopted as the primary methodology. These guidelines and designs are essential tools in antimicrobial stewardship, many studies have used these models and guidelines to optimise antimicrobial therapy (Dawaiwala et al., 2023).

### Study approval

The study was approved by the Hospital's administration. Since it was an exploratory study, no ethical approval was required. However, all procedures were followed following the ethical standards of the responsible committee on human experimentation and the Helsinki Declaration. Before data recording verbal consent was taken from each patient during the history-taking and data collection.

### Data collection

**Figure 1.**
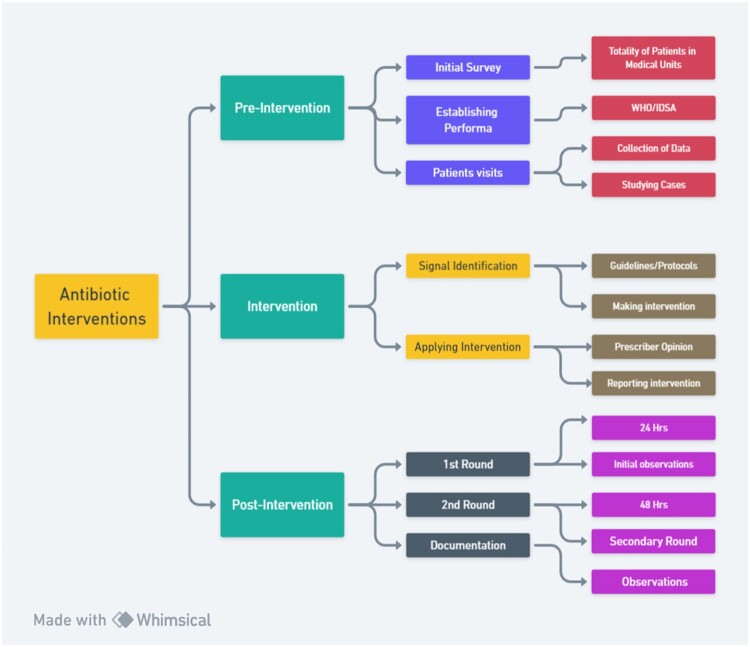
Display of methodology used in the study.

A structured questionnaire was established using the WHO Model to collect data. Patients were visited personally to collect data. Data was collected covering the general information about patients, including the patient's name, age, specific ward, date of admission, and weight. In the clinical section, information regarding primary and secondary diagnosis was noted. Additionally data regarding antimicrobial therapy: drug, dose, frequency, reason for selection, and duration of antibiotics. Patients’ lab reports were also checked and noted to compare with drug use guidelines. Due to the unavailability of microbiological culture data in most of the patient profiles, so only the available resources were used. All patients participated in data collection procedures; patient profiles were available at the patient bed desk, and nursing monitoring room, moreover ward's in-charge pharmacist and charge nurses also assisted in data collection (Figure 1).

### Patient selection criteria

The target population consisted of infectious disease patients who had received antibiotic therapy. Patients who were receiving antibiotic treatment on an ongoing basis; older than 18 years old were included in the study. Data was categorised according to the patient's infections, such as gastrointestinal infections, and listed accordingly. To ensure the study's rationality, patients with comorbidities were excluded. To collect data in the meantime, a time-based and convenient sampling approach was used (Figure 1).

### Interventions

Interventions included the modification and optimisation of antibiotic therapy according to guidelines following patient assessment and signal identification. WHO Antimicrobial Therapy, Society of Infectious Diseases Pharmacists (SIDP), and ASHP's book The *Pharmacist's Guide to Antimicrobial Therapy and Stewardship* were among the guidelines. The actions were based on basic principles of optimisation, which included identifying signals in patient profiles, assessing appropriateness and inappropriateness, checking for dose and dispensing errors, addressing therapeutic compliance, implementing therapeutic interchange, addressing adverse events, avoiding therapeutic duplication, and adjusting doses. These actions were then compared to guidelines, discussed with the prescriber about potential optimisation, and appropriate actions were then taken. In intervention procedures, a detailed analysis of the patient's profile was conducted during their visit and identified potential signs. The process involves re-evaluating and thoroughly examining the case, followed by a post-case conversation with the prescriber regarding the signal. Following a thorough discussion, the patient received an intervention following the guidelines. Patient outcomes were monitored after 24 and 48 h post-intervention (Figure 1).

### Post-interventions follow-ups

Two follow-up visits were conducted, one after 24 h and the second after 48 h, according to the WHO guidelines, and we evaluated patient outcomes and presented them to the prescriber (Figure 1).

### Data analysis

After data collection procedures, data was analyzed using GraphPad Prism (Version 9.0.2) and Microsoft Excel 2021. Chi-Square was applied to the interventional data to assess the statistical difference. Following the assessment, data was organised into graph formats for inclusion in the document. Whimsical and Microsoft PowerPoint 2021 was used for the graphical presentation of charts and figures.

## Results

### Demographics

**Figure 2.**
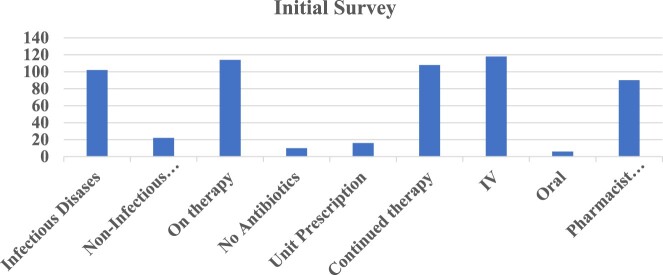
Results of the initial survey.

During the assessment period, a total of 124 patients were enrolled, out of which 102 received total antimicrobial therapy. An initial survey was conducted before the study to identify the disease and determine the type of therapy. In the initial survey, 102 patients were identified with infectious diseases and 22 patients with other diseases and local ailments, such as fever of unknown origin, poisoning cases, prophylaxis, etc., all of whom received one antibiotic dose (Figure 2). For the route of administration, 118 patients were on IV, and only 6 patients were on oral therapy. Overall, pharmacists from different medical units reviewed approximately 90 patients (Figure 2). The following table shows different demographics of patients, including totality and variation in different diseases. Among patients, the mean age was 45.6 before the intervention and 44.5 after the intervention. Out of the 124 patients, 78 were male and 46 were female. Out of all the cases 102 selected patients with infectious diseases were categorised into Urinary Tract Infections (*n* = 24), Respiratory Tract Infections (*n* = 22), Bloodstream Infections (*n* = 26), Intra-abdominal Infections (*n* = 22), Skin and Soft Tissue Infections (*n* = 5), and Central Nervous System Infections (*n* = 13), with the remaining 12 patients including non- and other ailments (*n* = 12) (Table 1).

**Table 1.** Demographics of patients.

General characteristics of patients in pre and intervention periods			
Demographics	Pre- intervention *n*, %	Intervention *n*, %	*p*-value
Total patients	124	102	
Age (mean)	45.6	44.5	0.83
Male	78	65	0.24
Female	46	37	0.27
**Primary source of infection**			
Urinary tract infections	24 (19.35)	17 (16.66)	0.21
Respiratory infections	22 (17.74)	22 (21.56)	>0.99
Blood stream infections	26 (20.96)	23 (22.54)	0.77
Intra-abdominal infections	22 (17.74)	18 (17.64)	0.52
Skin and soft tissue infections	5 (4.03)	5 (4.90)	>0.99
Central nervous system infections	13 (10.48)	10 (9.80)	0.98
Others (fever of unknown origin, etc.)	12 (9.67)	7 (6.86)	0.35

### Interventions

During the intervention period, 136 interventions were offered, of which 66% (90 interventions) were accepted. The remaining interventions were dismissed as unjustified because of insufficient drug supply and favourable feedback from the prescribers. Among the accepted interventions, 12 out of 17 (70.5%) were due to excessive use, while 11 out of 14 (78.5%) were related to irrelevant spectrum. Thirteen interventions were conducted for dose adjustments following WHO and IDSA guidelines on antibiotic use. Additionally, 7 interventions (63.6%) were made for dose frequency adjustments. A significant number of interventions, totalling 21 (70%), were related to spectrum-based antibiotic selection. Furthermore, 6 interventions addressed therapeutic duplication, 9 involved double microsomal coverage, 6 were for discontinuation due to lack of indication, 4 antibiotics were switched from intravenous to oral administration, and 1 was discontinued due to suspected adverse drug reactions. The investigation of intervention categories and their associated acceptance rates offered insights into clinical decision-making during the intervention period. Interventions aimed at de-escalation due to excessive usage and spectrum received significant acceptance, demonstrating their adaptability to the therapeutic setting. According to established criteria, the 86.6% acceptance rate for dosage modifications demonstrates the efficacy of following prescribed methods. The classification of antibiotic selection according to spectrum demonstrated a significant acceptance rate, underscoring the need for tailored treatment strategies. On the other hand, categories such as IV to oral changeover and probable adverse drug reactions had lower acceptance rates, indicating possible obstacles or complications within these specific intervention domains. Notwithstanding the fluctuations in acceptance rates within categories, the aggregate success rate of 66.17% for approved treatments evidenced the efficacy of the intervention techniques in enhancing patient care and antibiotic consumption (Table 2).

**Table 2.** Data of interventions.

Categories of interventions and their accepted rate			
Type of intervention	Proposed *n*, %	Accepted *n*, %	*p*-value
De-escalation because of excessive use	17 (12.5%)	12 (70.5%)	0.30
De-escalation because of the spectrum	14 (10.3%)	11 (78.5%)	0.54
Dose adjustment as per standard guidelines	15 (11%)	13 (86.6%)	0.70
Dose frequency adjustment	11 (8%)	7 (63.6%)	0.34
Antibiotic selection as per spectrum	30 (22.05%)	21(70%)	0.16
Therapeutic duplication	9 (6.6%)	6 (66.6%)	0.60
Double microorganismal coverage	15 (11.02%)	9 (60%)	0.22
Discontinuation for no indication	9 (6.6%)	6 (66.6%)	0.30
IV to oral switch	11 (8.08%)	4 (36.3%)	0.03
Suspected ADRs	5(3.67%)	1 (20%)	0.10
Total	136 (99.82%)	90 (66.17%)	

## Use of antibiotics

Prescribers used a variety of antibiotics during the assessment, including both prescribed and restricted ones. The data below illustrates the percentage of all antibiotics used during this time.

Ceftriaxone utility (54 pt.) saw significant usage due to its widespread use against various potential infections and prophylaxis. They used vancomycin (30 pt.), meropenem (26 pt.), moxifloxacin (24 pt.), and metronidazole (22 pt.) in a higher ratio among other antibiotics. Statistically significant changes were observed in the usage percentages of antibiotics before and after the intervention for ceftriaxone, meropenem, moxifloxacin, and vancomycin (*p* = 0.0017, *p* = 0.0077, *p* = 0.016, and *p* = 0.015, respectively). Ceftriaxone usage decreased from 54% to 26%, while meropenem, moxifloxacin, and vancomycin usage decreased from 26% to 10%, 24% to 10%, and 30% to 14%, respectively. Some antibiotics, like gentamycin, metronidazole, azithromycin, cotrimoxazole, amoxicillin and clavulanic acid, ciprofloxacin, piperacillin, and tazobactam, doxycycline, vibromycin, showed different levels of changes in how they were used, with *p*-values showing changes that were not statistically significant. These results suggest that the intervention has a noticeable impact on the prescription patterns of certain antibiotics, necessitating further investigation into the underlying clinical factors driving these changes. Antibiotics were classified using the AWaRe Antibiotics Classification by WHO. This classification is a new norm to address the appropriate use of antibiotics. AWaRe classification divides antibiotics into three major classes, i.e. Access Antibiotics, Watch Antibiotics, and Reserve Antibiotics (Sulis et al., 2022). Access antibiotics possess a limited range of action, reduced cost, a favourable safety profile, and often negligible resistance potential. Physicians often recommend them as empirical first- or second-line therapeutic choices for prevalent illnesses. They only recommend Watch antibiotics as first-line treatments for patients exhibiting severe clinical manifestations or for infections where the responsible pathogens are likely to exhibit resistance to Access antibiotics. Watch antibiotics are broad-spectrum agents, typically more expensive. To address multidrug-resistant illnesses, reserve antibiotics are the last resort like Meropenem (Table 3).

**Table 3.** Data of use of antibiotics.

Use of Antibiotics in different patients			
Antibiotics	Pre- intervention *n*,	Post-intervention *n*,	*p*-value
**Access antibiotics**			
Ceftriaxone	54	26	0.0017
Amoxicillin and clavulanic acid	10	4	0.108
Azithromycin	10	6	0.317
Doxycycline	10	5	0.300
Gentamycin	10	6	0.317
Metronidazole	22	12	0.086
Vibromycin	10	6	0.317
**Watch antibiotics**			
Ciprofloxacin	15	8	0.210
Clotrimazoles	10	5	0.300
Piperacillin and tazobactam	10	3	0.095
Moxifloxacin	24	10	0.016
Vancomycin	30	14	0.015
**Reserve antibiotics**			
Meropenem	26	10	0.0077

## Discussion

Antimicrobial Stewardship Approaches have been very useful in reducing the inappropriate use of antibiotics and optimising the therapy. Our study also presents a successful attempt to address the inappropriate use of antibiotics and stewardship tools to optimise the issue. In the Asian region, Researchers from India published a paper explaining the same methodology for antimicrobial stewardship. He explained how interventions could be a leading assessment tool for managing antibiotic consumption (Dawaiwala et al., 2023). Individuals in Pakistan have conducted several studies on AMR and AMS, including a multicenter qualitative study on physicians’ knowledge, attitudes, and practices regarding AMS (Khan et al., 2020) and a study in which the authors discussed the need for defense, guidelines, and AMS in pre-and postoperative surgical practice policies to reduce the risk of developing surgical site infections (Atif et al., 2021). Our study focuses on how clinical pharmacists manage antibiotic therapy for patients with critical infections. The study's results demonstrate the critical need for interventions during therapy, as evidenced by the 66.17 percent acceptance rate of interventions, which is significantly high and validates the hypothesis. Furthermore, patients receiving interventions demonstrated positive outcomes. Remaining 33. 83% of interventions were rejected and remained under discussion; reasons for rejecting those interventions were prescriber’s arguments against given antibiotics, trouble intervening according to the signal, superinfections, mixed spectra, and several others. The acceptance of an intervention was contingent upon the prescriber's opinion and the patient's condition. While some prescribers expressed reluctance to accept given interventions due to their patients’ severe conditions and diagnosis of superinfections, others advocated for the de-escalation of dose and drug to ensure patient safety. The examination of antibiotic use explains the prescribing pattern for antibiotics for critical care patients. Most of the prescribers were prescribing Access antibiotics like Ceftriaxone with regular doses; at this point, de-escalating the dose was the leading factor in preventing patient criticality. Patients with local and severe systemic infections used Watch and Reserve antibiotics such as Meropenem, Moxifloxacin, and Vancomycin, with a focus on adjusting the dose and selecting a specific drug of choice. Optimal antibiotic use is associated with collecting cultures before administering the antimicrobial agent (Owens et al., 2012). In our study, most patients didn’t have appropriate culture studies to support targeted therapy of antibiotics, which is a major regressing factor. That’s why the inclusion of microbial cultures was ruled out. However, this is a concerning issue that requires attention; prescribing an antibiotic to critical patients without checking cultural reports is heavily contributing to resistance.

This study implicitly examines stewardship practices in Pakistan, as there are a limited number of studies using prospective before and after optimisation. A retrospective optimisation study shows how important stewardship programmes are for reducing the overuse of antibiotics in tertiary care facilities (Bin et al., 2015). While addressing a physician's perspective, a study explores how clinicians feel about antimicrobial stewardship programmes in hospitals, which adds to the need for structured AMS interventions in Pakistan's healthcare system (Hayat et al., 2019). So, our study provides a pathway to prospective optimisation of antibiotic therapy in developing countries, especially Pakistan. In the future, this study has the potential to establish AMS at hospital levels and enhance it by incorporating outcome metrics such as DDD or DOT to optimise antibiotic therapy. This study employs all necessary stewardship tools, but in the future, multiple guidelines, such as the AWaRe Handbook of Antibiotic Optimization by WHO, can serve as supportive tools.

### Limitations of the study

The study has a precise approach to assessing the use of antibiotics and formulating interventions. The study has limitations to dose adjustments for renal and hepatic systems, antimicrobial resistance patterns, and long-term use of antibiotics. The study excluded microbiological culture reports due to data unavailability and did not use DDD and DOT outcome metrics.

## Conclusion

This study demonstrates that clinical pharmacy services may significantly improve antibiotic prescription practices in infectious disease departments. Our findings reveal a substantial reduction in inappropriate antibiotic use, accompanied by a corresponding improvement in patient outcomes. This explains the crucial role that pharmacists play in optimising antibiotic therapy, particularly in managing infections with a potential for antimicrobial resistance (AMR). The study highlights that the integration of clinical pharmacists in antimicrobial stewardship practices can significantly reduce inappropriate use of antibiotics and incidents of AMR.

## Supplementary Material

AMS Data Collection Performa.pdf
