# Supplementary material for: Pharmacist-led antibiotic interventions in infectious disease patients: a Pakistani tertiary care antimicrobial stewardship study
Source: J Pharm Policy Pract. 2025 Jan 16;18(1):2450017. doi: 10.1080/20523211.2025.2450017 (PMC11740292; doi:10.1080/20523211.2025.2450017)
Supplement: AMS Data Collection Performa.pdf [file JPPP_A_2450017_SM0008.pdf]

AMS Review Form:

DEPARTMENT:	CASE NO:

PATIENT DETAILS:		
Date:	Age:	Ward:
Name:	Sex:	Weight:

CLINICAL DATA:	
Major Diagnosis:	
Other Diagnosis:	
Type of Infection:	
Type of Therapy:	
Previous Therapy:	

MICROBIOLOGICAL DATA:	
Microbiology Sample:	
Isolated causative agent:	
Sensitivity to Antimicrobials:	
Date of Collection:	

ANTIMICROBIAL PRESCRIPTION:				
Drugs	Dose:	Route:	Interval:	Start Date

Ali Hassan PharmD

**INITIAL REVIEW:**		
Is Indication for Antibiotic treatment documented?<br>☐ Yes<br>☐ No	Is Antibiotic Prescribed according to guideline?<br>☐ Yes<br>☐ No	Comments (If No):
Correct Dose:<br>☐ Yes<br>☐ No	Appropriate Route:<br>☐ Yes<br>☐ No	Treatment duration/Date?<br>☐ Yes<br>☐ No

**48 Hrs REVIEW:**				
☐ Is antibiotic treatment reviewed?		What action?		
☐ Escalate	☐ Continue	☐ De-Escalate	☐ Stop	☐ IV-Oral Switch
Comments for continuation:				
Condition of Infection:				

**Prescriber's Comments:**
**Clinical Pharmacist's Comments:**
**Clinical Microbiologist's Comments:**
**ID Nurse' Comments:**
**Student's Comments:**
